# Long-Term Bridge Training Induces Functional Plasticity Changes in the Brain of Early-Adult Individuals

**DOI:** 10.3390/bs14060469

**Published:** 2024-05-31

**Authors:** Bingjie Zhao, Yan Liu, Zheng Wang, Qihan Zhang, Xuejun Bai

**Affiliations:** 1 Key Research Base of Humanities and Social Sciences of the Ministry of Education, Academy of Psychology and Behavior, Tianjin Normal University, Tianjin 300387, China; 2 Faculty of Psychology, Tianjin Normal University, Tianjin 300387, China; 3 Institute of Sports Science, Tianjin Normal University, Tianjin 300387, China; 4 Inner Mongolia Mental Health Center, Brain Hospital of Inner Mongolia Autonomous Region, Hohhot 010000, China; 5 School of Psychology, Inner Mongolia Normal University, Hohhot 010000, China

**Keywords:** bridge expertise, perception advantage, mathematical processing, functional plasticity

## Abstract

The aim of this study was to investigate the impact of extended bridge expertise on rapid perceptual processing and brain functional plasticity in early adulthood, utilizing functional magnetic resonance imaging (fMRI). In this investigation, we compared 6 high-level college bridge players with 25 college students lacking bridge experience, assessing their intelligence and working memory. Additionally, we scrutinized behavioral performance and whole-brain activation patterns during an image perceptual judgment task. Findings indicated significant group and interaction effects at the behavioral level. Bridge players exhibited prolonged reaction times and enhanced accuracy on card tasks. At the neural level, the activation level of bridge players in the occipital lobe exceeded that of ordinary college students, with more pronounced group effects in the motor area and inferior parietal lobule during card tasks. This implies that bridge expertise in early adulthood induces functional plasticity changes in regions associated with visual processing and automated mathematical computation.

## 1. Introduction

Bridge, a four-player card game played in pairs, is also an intellectual activity involving various cognitive processes [1,2,3]. Similar to experts in other mind sports, studies have shown that bridge experts demonstrate consistent advantages. For instance, in tasks involving card reconstruction at various experience levels, experts observed the cards less frequently and for shorter durations than novices [4]. Furthermore, studies indicate that the average bidding latency, accuracy, problem-solving time, and overall performance of bridge players are all influenced by their skill levels. In comparison to novices, bridge experts demonstrate higher accuracy and quicker responses [2,4,5]. The studies mentioned above suggest that the perception and problem-solving abilities of bridge experts improve with training. However, some researchers have discovered that bridge life masters perform less proficiently than novices in perception tasks, necessitating more time for encoding and reconstruction and displaying lower efficiency [4]. Hence, further investigation is needed to explore the existence of expertise advantages in bridge.

While there is no consistent conclusion regarding the long-term training benefits on individual behavioral performance in bridge, and an in-depth exploration of the underlying mechanisms of these performance gains is still lacking, other experts in mind sports have yielded more consistent results and offered insights into the underlying mechanisms. According to chunk and template theory, experts can swiftly retrieve pertinent actions from memory through pattern recognition [6,7]. Experts match the current stimulus with patterns stored in long-term memory (LTM), thereby facilitating the retrieval of solutions. The advantages of experts in perception and automated problem-solving are emphasized. Like bridge experts, experts in other mind sports also demonstrate a perceptual advantage. This implies that experts possess a broader visual span, swiftly identifying target areas through pattern recognition, completing tasks with fewer fixations [8,9,10,11,12,13,14,15], and automatically processing stimuli [16]. The majority of researchers posit that the perceptual advantage forms the foundation for the expertise exhibited by experts in mind sports [6,13,16,17,18,19]. Its emergence can be attributed to the accumulation of a substantial amount of chunk/template information through long-term training in the field. This information allows experts to match current stimuli with chunks and employ automatically activated chunk information to accomplish related tasks. The face-specificity hypothesis suggests that the fusiform gyrus (FFA) is a region responsible only for processing faces [20,21,22], while the expertise hypothesis suggests that this region is a visual processing area that is regulated by experience and is not only related to face processing [23,24,25]. Therefore, FFA has aroused extensive research in the field of experts. The relevant brain imaging results support the expertise hypothesis that chess experts cause a high level of FFA activation when dealing with domain-specific stimuli compared to other stimuli. It is concluded that the FFA is the neural basis for the perceptual advantage shown by experts [26,27,28].

Apart from perceptual advantage, some researchers have examined experts in automated problem-solving processes. Pioneering researchers investigated this by examining problem-solving under time pressure. The results indicated that experts’ performance was relatively stable and less influenced by time pressure [12,29]. Subsequent studies discovered that experts continued to demonstrate superior performance in tasks requiring the processing of object relations and the automatic processing of abstract relations between objects [14,23,30]. The supramarginal gyrus (SMG) may be the brain region associated with automatic processing. For instance, Bilalic et al. [30] observed that the activation level of the SMG was higher in chess experts than novices during a threat task involving the processing of abstract chess piece relationships (judging whether black had four possibilities to capture white in a given position). However, no differences were noted between chess experts and novices in this area during a visual search task that did not involve the processing of chess piece relationships [9]. Moreover, certain researchers have identified the caudate nucleus as the brain region associated with automatic processing in shogi experts [31,32].

However, there remains controversy regarding the perceptual advantage and automatic processing of experts. Some researchers have failed to find evidence of a perceptual advantage in experts, indicating that there were no significant differences in average gaze time and proportion between experts and novices when completing threat tasks and check-detection tasks. In fact, experts exhibited longer fixation times and shorter saccade lengths than novices [18,30]. Consequently, the presence of a stable perceptual processing advantage for bridge experts requires further exploration.

Currently, research on automation primarily focuses on chess experts, but there are differences in the emphasis of experts in different fields. This leads to significant inconsistencies in the brain regions associated with automated processing. Although both chess and Chinese chess are board games that rely on the arrangement of pieces to solve problems, they have significant differences in rules and the roles of pieces compared to international chess. In contrast, bridge is a card game with lower spatial requirements, where the placement of cards has a smaller impact. Furthermore, unlike intellectual sports such as chess or shogi, which mainly involve abstract relationships of offense and defense, bridge primarily requires cognitive calculation skills. In bridge, players must bid based on the point values in their hands before playing a card. The parietal lobe is the hottest region of mathematical processing, and damage to this region can lead to acalculia [33]. The study found that compared with the non-mathematical tasks, the calculation task activates the parietal lobes [34,35,36]. Compared with algebraic computation, the inferior parietal lobules are also activated by calculation tasks [37], regardless of whether the calculation task is addition or subtraction [38]. TMS was used to stimulate the parietal lobe region, and it was found that this region had an impact on both response time and accuracy of arithmetic tasks [39,40]. In addition, children with mathematical disabilities showed increased brain activation in the frontoparietal network after training [41]; the abacus training group had different activation in the inferior parietal lobules than the untrained group [42]. Therefore, we hypothesize that prolonged bridge training would lead to plasticity changes in the brain regions responsible for mathematical processing.

Prior studies have frequently employed a broad age range for experts, and in some instances, only male experts were selected [9,23,27,43,44,45]. Numerous studies have identified long-term plasticity in the brain [46,47,48,49,50,51,52], and this plasticity exhibits non-linear changes with training time [53,54,55]. Furthermore, gender can exert an influence on neural activity and brain plasticity [56,57,58,59,60]. Therefore, controlling for the effects of development and gender on brain plasticity is crucial to derive a stable effect of experience on brain function [61,62].

Considering the previous research deficiencies in participant gender, age range, and stimulus presentation, coupled with the fact that bridge training primarily occurs online, which can mitigate the masking effect of stimulus presentation methods on expert advantages, we conducted a functional magnetic resonance imaging (fMRI) study comparing the behavioral performance and neural activity of bridge players and typical college students in perceptual tasks. This aimed to validate the impact of bridge training on individual plasticity. With participants limited to early adulthood, we hypothesized that in processing domain-specific stimuli, bridge experts would demonstrate perceptual advantages and automated processing. Expertise would not only lead to stronger activation in the FFA [24,27,63,64], responsible for perceptual advantage, but also elicit robust activation in the parietal lobe, associated with automated mathematical processing [39,65].

## 2. Materials and Methods

### 2.1. Participants

Six elite bridge players were recruited from the Tianjin Normal University team, commencing their bridge training in early adulthood. They averaged a training period of 4 years and 9 months, with a weekly training frequency of 15 h. In total, 4 out of 6 bridge players won the 2020 FISU WUC Mind Sports Online Championship. Among them, 1 out of 3 holds the title of Regional Master, 1 out of 3 holds the title of Club Master, and 1 out of 3 holds the title of Grand Master for Life. The time taken to achieve these current levels ranges from six months to two years. Twenty-five ordinary college students were matched with the bridge players in terms of intelligence quotient (IQ) and working memory (WM).

All participants were right-handed, had normal or corrected-to-normal vision, were free from implanted metal, exhibited no claustrophobia, and had no history of psychiatric disorders, neurological diseases, or addictive behaviors. Informed consent was secured from all participants, and the study received approval from the Ethics Committee of the Academy of Psychology and Behavior at Tianjin Normal University. Upon completion of the experiment, participants were remunerated with monetary compensation.

Table 1 presents the demographic information for both groups. The results of the non-parametric Mann–Whitney U test indicated significant differences between the two groups in age and years of education (Zs < −2.81, ps < 0.05), while no significant differences were observed in IQ and WM (Zs > −1.85, ps > 0.05). Therefore, in the subsequent analysis, age and years of education were included as covariates for both groups of subjects.

### 2.2. Questionnaire

The Chinese version of the Combined Raven’s test [66] was employed to assess IQ. This test comprises exclusively graphical problems, prompting participants to select one of six to eight answers to complete a provided image, ensuring logical and comprehensive solutions. Comprising six sets of twelve questions, this test progressively increases in difficulty. Participants are mandated to sequentially complete the test within a 40 min timeframe. Participants are required to complete the test in sequence within 40 min. Each question carries a one-point value, yielding a total of 72 points. A higher score reflects a heightened level of intelligence.

The Digit Span test, adapted from the Wechsler Adult Intelligence Scale [67], was employed to evaluate individuals’ WM. Widely utilized in numerous studies [68,69], this test has proven effective for assessing working memory. During this test, a series of numbers ranging from 1–9 is orally presented in random order, each number appearing for one second. The length of the series progressively escalates from two to eleven digits. Following the presentation of the series, participants are instructed to recall the sequence either forward or backward. Each series is presented three times, maintaining the same length but using different numbers. If the participant accurately recalls the sequence two times or more, the length of the series is incremented by one digit. The score for this test is determined by the length of the longest sequence recalled by the participant. A longer sequence signifies a higher level of WM. Scores range from a minimum of three points for forward recall and two points for backward recall to a maximum of eleven points for forward recall and ten points for backward recall. In our study, the WM indicator was derived from the sum of scores for both forward and backward recall.

### 2.3. Experimental Design

The design incorporated a between-group variable (group: bridge players, ordinary college students) and within-group variables (task: card judgment, shape judgment). Dependent variables encompassed response time (RT), accuracy (ACC), and neural activity during the task.

### 2.4. Stimulus

Bridge, an intellectual team sport, is played with a standard deck of 52 playing cards, excluding jokers. During the dealing stage, the dealer randomly distributes 13 cards to each of the four players. During the bidding stage, players employ predetermined bidding systems to exchange information with their partners, reaching agreements based on the point value of their hands and specific rules. This stage is pivotal for success in competitive bridge and represents the sole legal means for partners to communicate. It is also a distinctive feature of bridge. Considering the uniqueness and significance of this stage in bridge, the experimental stimuli utilized in this study were the card patterns presented during the bidding stage of a bridge game, comprising 13 cards. For the ordinary college student, the experimental stimulus is a picture of 13 cards; for bridge players, the experimental stimulus represents the familiar bidding stage of bridge. This domain of stimuli has more abstract semantic information.

Card Materials: Ensuring authenticity and ecological validity of the experimental materials, we randomly selected 13 cards from a standard deck of 52 playing cards (4 suits with 13 cards each) to generate 80 sets of card pattern stimuli. To simplify the stimuli’s complexity, card patterns were presented in a matrix. The first row displays the four suits (spades, hearts, diamonds, clubs) in a fixed order, with a 4 × 2 matrix below each suit representing the specific cards for that suit (AKQJ, 10-2). It should be noted that not all matrices under each suit contain information, given the limitation of 13 cards in the bidding stage.

Shape Materials: The fundamental elements of the shape materials correspond one-to-one with the spatial distribution of numbers in the card materials. The sole distinction lies in the shape materials comprising shapes randomly selected from six different categories, including semicircles, circles, triangles, squares, pentagons, and stars.

**Figure 1:**
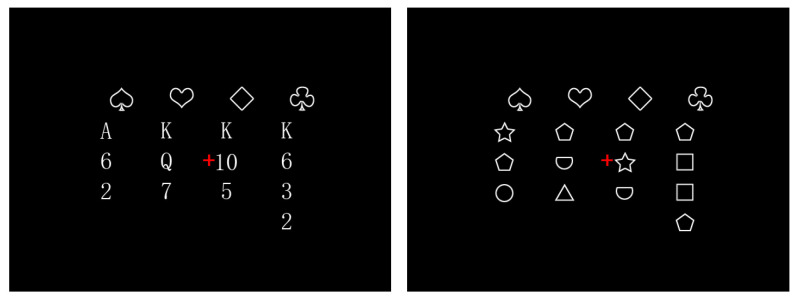
Experimental stimuli ((**left**): card material; (**right**): shape material). Note: The red plus sign was present throughout the experiment, which not only made it easier for individuals to focus on the center of the screen, but also reduced the interference of stimulus changes.

Considering that the complexity and spatial arrangement of experimental stimuli can impact brain activity during fMRI experiments, the stimuli were presented at a visual angle of 15° × 9°. The stimuli had a black background, with numbers and suits presented in white. To control for the initial fixation position, a red fixation point was presented at the center of each stimulus, ensuring that participants’ initial fixation was on the center of the screen when observing the information. Each type of material consisted of 80 stimuli, resulting in a total of 160 stimuli. Refer to Figure 1 for an example of the specific presentation materials.

### 2.5. Experimental Procedure

Practice Stage: To ensure participants had a precise understanding of the experimental tasks, they completed practice tasks in the preparation room (practice stimuli did not appear in the formal experiment). The practice tasks mirrored the formal experiment task, where participants judged whether the preceding and succeeding images were the same or different (half of the participants pressed 1 if they were the same and 2 if they were different; the other half pressed 2 if they were the same and 1 if they were different). Participants proceeded to the formal experiment when their accuracy rate exceeded 80%.

**Figure 2:**
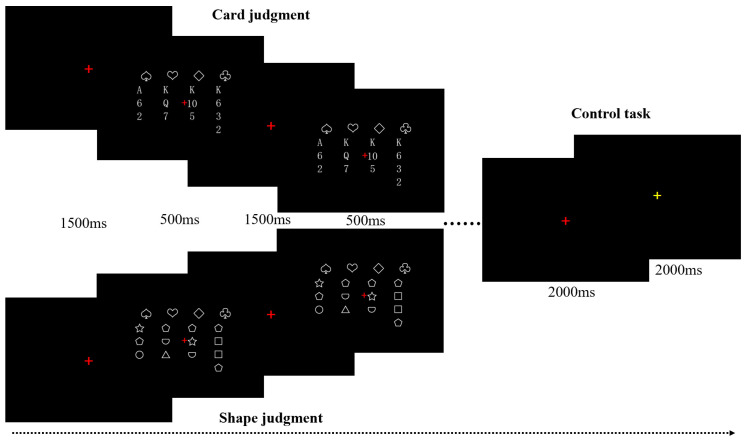
Overview of the experimental procedure in the MR scanner.

Formal Experiment: The formal experiment comprised tasks for card judgment, shape judgment, and button control. The card judgment task included four blocks, each containing 20 trials with card materials presented in a pseudorandom order. The experimental procedure for each trial was as follows: a red fixation point was presented for 1500 ms, followed by the card materials presented for 500 ms [70,71,72]. Participants were required to judge the consistency of the preceding and succeeding images as quickly and accurately as possible before the next card material appeared. The shape judgment task paralleled the card judgment task, except that the stimuli used were shape materials. The eight blocks for the card and shape judgment tasks were presented randomly, with a button control task of 12 s between each block. During the button control task, three red fixation points and three yellow fixation points were randomly presented, each lasting for 2 s. Participants were instructed to press the button quickly when the yellow fixation point appeared to eliminate the influence of the reaction button on the experimental results. The experimental procedure is shown in Figure 2.

### 2.6. fMRI Acquisition

All data were collected at the Tianjin Normal University Center using a Siemens Prisma 3.0 T scanner and a 64-channel head coil. The structural image was acquired using a magnetization-prepared rapid gradient echo sequence with the following scanning parameters: TR = 2530 ms, TE = 2.98 ms, flip angle = 7°, the resolution of each voxel was 1 mm × 1 mm × 1 mm, GRAPPA, phase-coded direction acceleration factor = 2, slices number = 192. The BOLD signal was acquired using the Echo-planar imaging sequence with parameters set as: TR = 2000 ms, TE = 30 ms, flip angle = 90°, slice thickness = 2 mm, slices number = 62, FOV = 224 mm × 224 mm, resolution of each voxel was 2 mm × 2 mm × 2 mm, parallel acquisition (multi-band), layer selection direction acceleration factor = 2, phase coding direction acceleration factor = 2.

### 2.7. Data Analysis

**Behavioral data analysis:** Due to the small sample size, we employed the function ‘aovp’ from the ‘lmPerm’ package in R (version 4.1.2) to conduct a 2 × 2 repeated-measures ANOVA with covariates. This analysis utilizes permutation tests instead of normal theory tests to calculate statistics [73,74,75]. In this scenario, the RT and ACC were extracted as dependent variables, with group and task as fixed factors, and age and education as covariates. The reported *p*-values are derived from permutation testing, with a significance level set at *p* < 0.05.

**fMRI data pre-processing:** The preprocessing was conducted using DPABI V4.2 (http://rfmri.org/dpabi) (accessed on 10 April 2024) [76] within the Matlab R2012a platform (http://www.mathworks.com/) (accessed on 10 April 2024).

It included the following procedures—(1) format: the raw data were converted into NIFTI format; (2) time point: due to the instability of the signal in the initial acquisition phase, the first six time points of data for each participant were removed, corresponding to the control task at experiment; (3) slice timing: as the experiment involved parallel scanning, we utilized SPM12 to determine the reference slice and correct the acquisition timing differences for different locations; (4) realignment: to correct for head motion of participants during the scan, any participants who moved more than 1.5 mm or 1.5° in any direction were excluded from further analysis; (5) normalization: DARTEL was employed for image normalization to mitigate the brain differences between individuals; (6) smoothing: spatial smoothing was applied using a Gaussian kernel with a 6 mm full-width at half-maximum (FWHM).

**Statistical fMRI analysis:** The analysis at the individual and group levels is primarily conducted in SPM12 (http://www.fil.ion.ucl.ac.uk/spm) (accessed on 10 April 2024). For group analysis, pTFCE (Threshold-Free Cluster Enhancement) [77,78] is incorporated. Additionally, ROI (Region of Interest) analysis is performed using DPABI and R.

(1) Individual-level analysis: After pre-processing, the image data of each subject were combined for further analysis. Single-subject analysis was conducted through linear regression on the 31 participants in three conditions: the card condition, the shape condition, and the baseline condition (button control). This was achieved by specifying the onset time of each trial in different conditions and using the six realignment parameters as covariates. After estimating the parameters, a design matrix was finally obtained for each subject, which was compared with the baseline condition under both card and shape conditions. (2) Group-level analysis: Following the individual-level analysis, an all-factor model was employed, with groups and tasks as fixed effects, while age and years of education were controlled as covariates. Multiple comparisons and correction of group-level analysis results generated by SPM were carried out using TFCE, to improve the stability and reliability of statistical results of non-parametric data. Statistically significant results were determined when *p* < 0.001 (after TFCE correction) and cluster size > 30. (3) ROI analysis: We used wfupickatlas to define ROIs with a radius of 6 mm based on the MNI coordinates of the differential brain regions obtained in the group-level analysis. Subsequently, SPM was employed to calculate the intersection of ROI and group analysis results, and the BOLD response of each ROI was extracted in DPABI. Similar to behavioral metrics, the extracted BOLD responses underwent repeated measures ANOVA with permutation in R, and significance was considered when *p* < 0.05. (4) Correlation analysis: Due to the limited number of bridge players, challenges arose in grouping and calculating correlations between behavior and brain regions based on participant categories. Therefore, a task-based grouping approach was adopted. Specifically, behavioral metrics and BOLD activation signal values were separately extracted for the card task and the shape task for all participants. Utilizing age and education as covariates, partial correlations were calculated between the differences in RT, ACC, and BOLD signals for the card and shape tasks.

**Figure 3:**
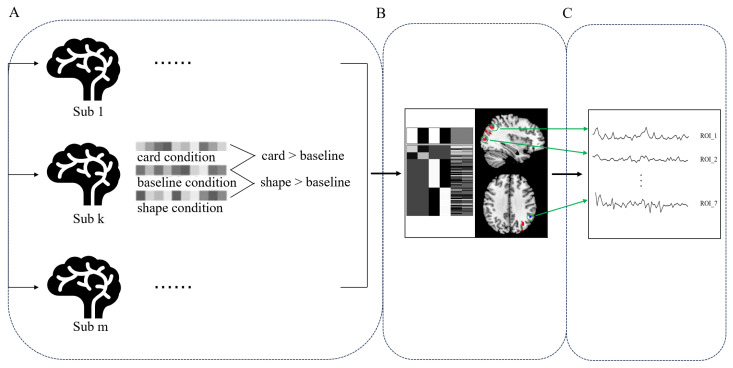
fMRI data analysis. (**A**) Individual-level analysis, calculating differential activation for each participant separately compared to baseline conditions in the card and shape conditions. (**B**) Group-level analysis, performing whole-brain variance analysis on data from all participants. (**C**) ROI-based analysis, extracting BOLD signals from regions of interest with differential activation.

The analysis process of fMRI data is shown in Figure 3.

## 3. Results

### 3.1. Behavioral Results

The group effect was significant on the index of RT (df = 1, mean square (MS) = 69,158, *p* < 0.05), while the task effect was also significant (df = 1, MS = 963.70, *p* < 0.05). However, the interaction effect was not significant (df = 1, MS = 1228.59, *p* = 0.159; in the card judgement task, *M*_bridge_ = 649, *M*_ordinary_ = 571, *SD*_bridge_ = 118, *M*_ordinary_ = 101; in the shape judgment task, *M*_bridge_ = 623, *M*_ordinary_ = 568, *SD*_bridge_ = 106, *M*_ordinary_ = 92).

**Figure 4:**
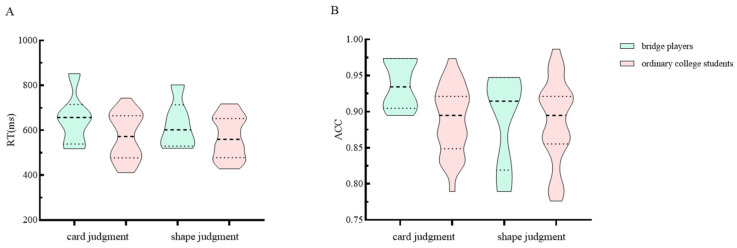
Behavioral results. (**A**) This panel shows the mean RT of the two groups of subjects under different tasks. Error bars indicate standard errors. (**B**) This panel shows the mean ACC of the two groups of subjects under different tasks. Note: The short and long dotted lines in the violin plot are the quartile and median, respectively.

Regarding ACC, the group effect was not found to be significant (df = 1, MS = 0.007, *p* = 0.294), but the task effect was significant (df = 1, MS = 0.002, *p* < 0.05), and the interaction effect was marginally significant (df = 1, MS = 0.005, *p* = 0.073). Further analysis of simple effects revealed that there were significant group effects in the card judgment task (*M*_bridge_ = 0.93, *M*_ordinary_ = 0.88, *SD*_bridge_ = 0.03, *M*_ordinary_ = 0.04, df = 1, MS = 0.017, *p* < 0.01), but not in the shape judgment task (*M*_bridge_ = 0.89, *M*_ordinary_ = 0.88, *SD*_bridge_ = 0.06, *M*_ordinary_ = 0.05, df = 1, MS = 0.0002, *p* = 0.804). The results of the behavioral data are shown in Figure 4.

We conducted separate correlational analyses on reaction times and accuracy rates for the two groups of participants to gain insight into their cognitive processing strategies during the task. The results revealed that for ordinary college students, there was a moderate positive correlation between reaction time and accuracy under the card judgment task (r = 0.512, *p* = 0.009), whereas under the shape judgment task, the correlation was negligible (r = 0.389, *p* = 0.055). Conversely, for the bridge players, the correlation between reaction time and accuracy was negligible under both the card and shape judgment task (rs < 0.516, ps > 0.05).

### 3.2. fMRI Results

#### 3.2.1. Whole-Brain Analysis

**Figure 5:**
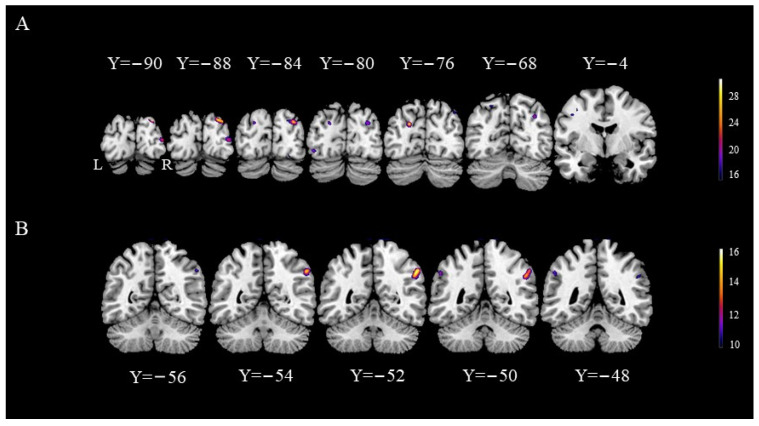
fMRI findings. (**A**) This panel illustrates the coronal plane of the brain where group effects are evident. In these areas, bridge players exhibit higher BOLD responses compared to college students, and the accompanying bar chart on the right represents the F-test scores. (**B**) This panel displays the coronal view of the brain where the task effect is observed, indicating a stronger BOLD response in the card task.

The results indicated a significant group effect, suggesting the presence of different activated brain areas between bridge players and ordinary college students. Compared with ordinary college students, bridge athletes exhibited higher activation in the bilateral superior occipital gyrus (SOG), left inferior occipital gyrus (IOG), right middle occipital gyrus (MOG), and left precentral gyrus (PCG). Significant task effects were observed in the right inferior parietal lobule (IPL). However, no brain regions with significant interaction effects were identified. Specific differences in the activation of brain regions are detailed in Table 2 and Figure 5.

#### 3.2.2. ROI Analysis

The BOLD response of brain regions exhibiting group effects was analyzed using permutation methods in R. The results revealed significant group effects in all brain regions (df = 1, MSs > 29.557, Ps < 0.007), indicating that compared with ordinary college students, bridge players exhibited higher BOLD responses in these regions. No significant task effects (df = 1, MSs < 1.231, Ps > 0.153) or interaction effects (df = 1, MSs < 0.073, Ps > 0.369) were observed in all brain regions except the left PCG (task effect: df = 1, MS = 0.145, *p* < 0.05; interaction effect: df = 1, MS = 0.228, *p* < 0.05). Further simple effect analysis indicated a significant group effect under both tasks, but the group effect under the card task was more pronounced (card judgment task: df = 1, MS = 6.600, *p* < 0.001, *M*_bridge_ = 1.47, *M*_ordinary_ = 0.37, *SD*_bridge_ = 0.58, *M*_ordinary_ = 0.47; shape judgment task: df = 1, MS = 3.028, *p* < 0.001, *M*_bridge_ = 1.13, *M*_ordinary_ = 0.33, *SD*_bridge_ = 0.70, *M*_ordinary_ = 0.44).

**Figure 6:**
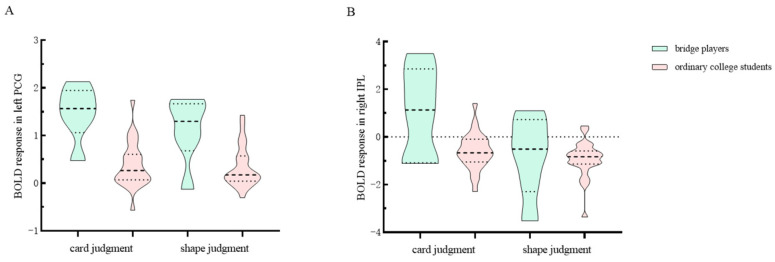
BOLD responses. (**A**) This panel illustrates the BOLD response of the left precentral gyrus (PCG) where interaction effects are observed. In this area, the card task induced a larger group effect. (**B**) This panel displays the BOLD response of the right inferior parietal lobule (IPL), where the group effect is significant in the card task. Note: The short and long dotted lines in the violin plot are the quartile and median, respectively.

Additionally, the BOLD response of brain regions with task effects was analyzed, and the results revealed significant task effects (df = 1, MS = 6.153, *p* < 0.001) and interaction effects (df = 1, MS = 5.311, *p* < 0.01) in right IPL. No significant group effects were observed in the right IPL (df = 1, MS = 4.349, *p* = 0.216). The simple effect results indicated a significant group effect in the card judgment task (df = 1, MS = 11.433, *p* < 0.01, *M*_bridge_ = 1.03, *M*_ordinary_ = −0.61, *SD*_bridge_ = 1.91, *M*_ordinary_ = 0.78), but not in the shape judgment task (df = 1, MS = 0.186, *p* = 0.491, *M*_bridge_ = −0.79, *M*_ordinary_ = −0.95, *SD*_bridge_ = 1.69, *M*_ordinary_ = 0.76). See Figure 6 for details

#### 3.2.3. Correlation Analysis

**Figure 7:**
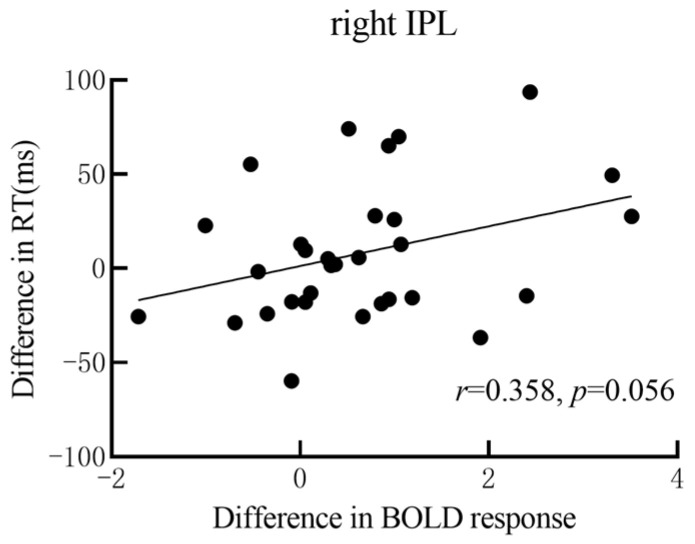
Correlation between behavior and BOLD response in the right inferior parietal lobule (IPL). The *y*-axis represents the difference between response time (RT) in the card task and RT in the shape task (RTcard—RTshape), and the *x*-axis represents the difference between BOLD responses in the right IPL evoked by the card task and BOLD responses in the shape task (BOLDcard—BOLDshape).

The results of partial correlation found a significant correlation between response time differences and BOLD signal activation differences in the right IPL (r = 0.358, *p* = 0.056). No correlation was observed between task-induced behavioral differences and BOLD response differences in other brain regions. See Figure 7 for details.

## 4. Discussion

Building upon prior research, this study utilizes fMRI technology to investigate the impact of bridge expertise on the plasticity of both external behavior and internal brain functions in early adulthood. Results reveal that at the behavioral level, irrespective of the judgment task, the RT of the bridge group is significantly higher than that of the control group. However, only in the card task, the ACC of the bridge group exceeds that of the control group. At the neural level, group effects are observed in the occipital lobe and left PCG; the task main effects are associated with the right IPL, and their interaction effects are primarily manifested in the PCG and IPL. Task-induced IPL activation differences were positively correlated with response time differences.

### 4.1. Expert Advantage in Bridge

In this study, it was observed that experts demonstrate a higher proficiency in processing domain-specific stimuli. Specifically, in the card task, bridge players exhibit greater ACC, whereas in the shape task, there is no significant difference in ACC between the two groups. Comparable findings have been reported in the realm of intellectual expertise [43,45,72]. For instance, chess experts display heightened accuracy only in real-game scenarios compared to novices, with no significant differences in other stimulus materials, and in some cases, they may even perform slightly worse. This outcome suggests that expertise equips experts with in-depth domain-specific knowledge, enabling them to effectively leverage rich knowledge structures and achieve higher accuracy in tasks. It is crucial to highlight that this behavioral advantage is constrained to the specific domain and does not extend to stimuli outside the domain. This indicates that the perceptual processing advantage resulting from expertise in bridge is confined to domain-specific stimuli and does not lead to a general change in perceptual abilities.

However, in terms of RT, we observed that bridge players require a longer RT to complete tasks, even within the domain-specific tasks. This finding contradicts the results of most expert–novice paradigms, where experts typically exhibit faster RT during task completion [15,23,79]. Yet, the phenomenon known as the expertise reversal effect has also been identified in bridge experts and experts from other domains, indicating that experts may perform worse under certain conditions [4,11,80,81]. The reasons for this result may lie in the different cognitive processing strategies employed by the two groups of participants. This viewpoint is supported by the results of correlation analysis, wherein significant correlations were found between RT and ACC in the ordinary college student group under the card task, whereas the correlations were not significant in the bridge players’ group. This result suggests that compared to bridge players, ordinary college students indeed require a longer time to ensure higher accuracy in the task; however, the completion time for the expert group is not related to accuracy, implying that bridge players engage in additional processing. In contrast to ordinary college students, the card task holds additional domain-specific meaning for bridge players, leading them to engage in deeper semantic processing of card stimuli. However, in this experiment, strict reliance on domain-specific knowledge is not necessary; participants can complete the task through general selective attention and memory. This also highlights the conditional nature of expert advantages—when domain-specific knowledge aligns with task requirements, expert gains are more likely, but when experts encounter a large volume of visual stimuli, it can introduce additional processing load, resulting in the expertise reversal effect [82].

In this study, we confirmed the existence of expertise effects through the ACC metric. The accumulation of expertise does indeed affect individuals’ cognitive performance [9,23,83,84]. However, we did not find a significant group effect on the ACC metric, whereas we observed an expert reversal effect on the RT metric. This is similar to the results of Guo et al. [85], which found that there was no significant difference in ACC between table tennis players and non-players, but there was a significant difference in RT, that is, athletes reacted faster than non-athletes during both types of the tasks. No distinction was detected in accuracy between the two groups’ behavioral outcomes. Both groups of participants exhibited high performance, indicating the seriousness with which individuals completed the task. Furthermore, it is possible that the competitive level of the experts in the laboratory setting was not stimulated as it might be in a competitive match, thus, there was no expert advantage. Meta-analysis results suggest that the existence of expert advantages is modulated by the type of expertise and the presentation of stimulus [86]. Compared to traditional physical sports that require bodily muscles and bones, with the mastery of motor skills as the primary goal, intellectual sports predominantly involve cognitive skills that require mental participation [87]. Thus, bridge players are more focused on the quality of decision-making, aiming to provide accurate answers within a given time frame. On the other hand, table tennis players need to react more swiftly, prioritizing response time. In addition, when the presentation of stimuli does not align with real-life scenarios, the advantage of experts might be obscured [19]. In summary, bridge players in this study exhibited longer response times and higher accuracy, reflecting a more profound and comprehensive processing during task completion.

### 4.2. The Reason of Expert Advantage

Previous researchers, utilizing brain imaging technology, primarily focused on investigating the internal mechanisms underlying the advantages of chess experts, with a particular emphasis on the role of the FFA. However, they overlooked the roles of other brain regions and the generalizability of the results to experts in other domains. Through whole-brain analysis of fMRI data, our study revealed significant differences in activation primarily in the occipital lobe between bridge players and ordinary college students. Compared to ordinary college students, bridge players exhibit strong activation in the occipital lobe, including areas such as SOG, MOG, and IOG, when performing tasks. This finding aligns with results in other expert domains [88,89,90]. Previous research has confirmed that the occipital lobe is a core brain region for visual processing [91,92,93], playing a crucial role in visual imagination, information integration, and maintaining visual attention, and memory [94,95,96,97,98,99]. This differential activation in the region indicates that expertise leads to functional reorganization of the brain. Experts develop highly generalized visual and attentional processing skills within their specialized fields. This means their visual system has been optimized to process various types of visual stimuli more efficiently, not just those specific to their domain. Another possibility is that experts are more familiar with shape stimuli, making them less affected by the type of stimuli presented.

Furthermore, we observed significant interaction effects in PCG and IPL, indicating higher activation levels in these regions for experts compared to ordinary college students during domain-specific tasks. As explained by Poldrack (2015) and Logan’s theory of automatization (1988), the way information is processed changes with the development of expertise in a specific field [100,101,102]. For ordinary students, the card condition merely required a numerical consistency judgment task; however, for bridge players, it additionally involved more abstract semantic processing automatically.

The PCG, located in the primary motor cortex, is associated with action-related language comprehension, action selection and implementation [103,104]. The increased activation in this region suggests that bridge players may automatically process stimuli according to bridge rules, such as card point values, bidding, and responding [102]. The IPL is primarily responsible for numerical computation, and meta-analytic results have shown that the IPL is crucial for numerical processing, comparison, and calculation [38,105,106,107,108,109,110,111]; expertise can lead to increased activation in this region [42]. The strong activation of the IPL indicates that bridge players automatically performed calculations related to the point value of the stimuli.

Most importantly, through correlation analysis, we found a positive correlation between IPL activation and RT, consistent with behavioral indicators. That is, bridge players with longer RT do not necessarily exhibit higher ACC. This result not only suggests that experts match current information with templates in LTM to obtain semantic information related to plans and tactics [112,113] but also indicates that there are indeed domain-specific differences in the automated processing brain regions among experts in different fields. Even among intellectual sports experts, specific expertise programs lead to different changes in brain functional plasticity. In conclusion, given the specificity of expertise, bridge players automatically engage in deeper semantic processing of stimuli within their domain during the card task, resulting in the widespread activation of math-related brain regions.

Finally, prior research suggests that the FFA is a specific brain region for face processing [20,114,115,116,117]. However, studies in expert domains have found that the FFA may also be involved in processing stimuli outside of faces. The researchers propose the expert experience hypothesis, suggesting that the FFA participates in the processing of any type of visual stimulus as long as the observer has accumulated a significant amount of visual knowledge and experience with the stimuli that need processing [23,24,25,27,28].

The role of the FFA in visual processing remains controversial. Studies on experts in areas such as cars and butterflies did not find differential activation in the FFA [20,21,26,118]. The activation of the FFA region did not show a significant correlation with the participants’ expertise level and judgment performance [21,119]. In some cases, experts even exhibited lower activation levels in the FFA compared to novices [45]. One possible reason for these results is that studies in expert domains often have insufficient sample sizes, leading to the instability of results [43,120]. This may also suggest that perceptual advantage is not key to the existence of expert advantages; rather, it is the flexibility in automated responses during tasks, driven by the memory acquired through expertise. Variations in the automation performance of different activities lead to the existence of expert advantages and specific manifestations that vary considerably [121].

## 5. Limitations and Deficiencies

Here, we demonstrate that bridge players had longer reaction times when processing domain-related stimuli, which may be due to the fact that although both groups were highly familiar with the experimental stimuli, only bridge players automatically activated responses in their field. Due to the limited number of bridge players, this study is only a pilot study, and its generalizability and the stability of its results may be insufficient. Further investigation with larger and more diverse samples would be more appropriate. In addition, in future research, eye-tracking technology can be used to explore the behavioral performance of experts and novices in perceptual tasks, while dynamic functional connectivity or multimodal analysis methods can be used to delve into the underlying neural mechanisms of bridge players and ordinary college students during this task from multiple perspectives.

## 6. Conclusions

This study found that expertise leads to individuals being more inclined to recognize chunks to complete tasks. When the task is purely perceptual, although chunks facilitate the process of perceptual encoding, the automatic processing of other abstract information in the LTM can prolong the expert’s response time. Moreover, prolonged training in bridge can cause plastic changes in areas responsible for visual processing and mathematical calculation.

## Figures and Tables

**Table 1:** Demographic information.

Group	Number	Male/Female	Age	Education Years	IQ	WM
bridge players	6	3/3	22.83 (1.94)	16.50 (1.38)	66.33 (3.88)	17.50 (2.59)
ordinary college students	25	10/15	20.52 (1.36)	14.76 (1.05)	63.72 (4.97)	15.40 (2.57)

Standard deviation is indicated in parentheses.

**Table 2:** Overview of fMRI activations. All x, y, and z values represent stereotaxic coordinates according to the coordinate system by the Montreal Neurological Institute (MNI) space. Statistical values correspond to the F-statistics and *p*-values of the activation maxima (peak voxel) within each anatomical region. For the contrast activations, the threshold was set at pTFCE < 0.001, with a cluster size > 30.

Region	Hemisphere	Voxels	MNI Coordinates			F Score
			x	y	z	
**Group effect**						
Occipital_Sup	left	73	−20	−76	28	29.118
Occipital_Sup	right	183	24	−88	34	27.793
Occipital_Inf	left	37	−42	−80	−6	19.413
Occipital_Mid	right	60	38	−90	8	22.209
Occipital_Mid	right	73	34	−68	38	21.451
Precentral	left	40	−42	−4	40	18.181
**Task effect**						
Parietal_Inf	right	34	52	−52	38	15.808

## Data Availability

The datasets presented in this article are not readily available because the data are part of an ongoing study. Requests to access the data set should be emailed directly to the corresponding author.
